# Providers’ views on hormonal family planning methods: a qualitative study from Dosso, Niger

**DOI:** 10.12688/gatesopenres.13674.1

**Published:** 2022-07-04

**Authors:** Amelia Maytan-Joneydi, Ellen W. MacLachlan, Balki Ibrahim Agali, Kyria Louis-Charles, Sanoussi Chaibou, Souleymane Amadou Garba, Abdoul Nouhou Moumouni, Ilene S. Speizer

**Affiliations:** 1 Carolina Population Center, University of North Carolina at Chapel Hill, Chapel Hill, NC, 27516, USA; 2 Independent Consultant, Seattle, WA, 98115, USA; 3 GRADE Africa, Niamey, Niger; 4 Maternal and Child Health, Gillings School of Global Public Health, UNC, Chapel Hill, NC, 27516, USA

**Keywords:** Niger, sub-Saharan Africa, family planning, counseling, method preference, provider bias

## Abstract

**Background:** Family planning (FP) providers play an important role in ensuring that clients are offered a full range of FP methods. This qualitative study explores providers’ views on three hormonal FP methods and why they think women may choose these methods in Niger.

**Methods:** In-depth interviews were conducted with 24 FP providers in 24 government health centers in Dosso region, Niger between February-March 2020. Providers were asked about the suitability of different FP methods for women, including adolescents and married women with children. The interviews were translated and transcribed from Hausa and Zarma into French, thematically coded, and qualitatively analyzed.

**Results:** Many providers believed discretion to be the most important method attribute for women. Providers report preferring implants for young clients because of the more rapid return to fertility. They disagreed on whether implants or injectables are more discrete for clients. That said, providers felt that clients appreciate the implant’s discretion, effectiveness, long-acting nature, and simplicity of use.  Providers perceived that the majority of women choose injectables due to familiarity with the method, the fact that it is “invisible” to an outsider, and a lack of awareness of implants. Providers stated that while women may not initially choose the implant, when given more information about it, they were more open to adopting it, or switching from another method, and less likely to believe local myths. Providers believed that women find pills to be indiscreet.

**Conclusions:** The findings highlight that while providers have perspectives on suitable methods for certain women, they also recognize that clients have their own preferences, such as how discreet the method is. As programs continue to expand method choice and new contraceptive technologies undergo research and development, highly desirable features such as discretion need to be considered.

## Introduction

Niger, a landlocked country in the Sahel region of West Africa, had a total fertility rate of 7.6 at the time of the last Demographic and Health Survey in 2012 ( Institut National de la Statistique (INS) et ICF International, 2013). Family planning (FP) use is not common in Niger, with 15% of all women, and 18% of married women reporting use of a modern method in 2017 ( PMA2020, 2018). Compared to married women, fewer married adolescents aged 15–19 years (11%) and married women with 0-1 children (13%) reported use of a modern method of FP ( PMA DataLab, 2021). In 2017, 21% of married women had an unmet need for FP for limiting and spacing pregnancies, that is, they reported a desire to delay or avoid pregnancy but were not using a method of contraception, putting these women at risk for unintended pregnancy ( PMA2020, 2018). Since early in the FP2020 (now FP2030) initiative, the government of Niger has made commitments to increase budg *et al*locations for FP and to increase contraceptive use through task sharing between health care worker cadres and including injectables in the basic service package offered by community health workers ( Family Planning 2020, 2021). Niger is also a member of the Ouagadougou Partnership and Sahel Women’s Empowerment and Demographic Dividend project (SWEDD), two large initiatives that provide funding to Niger with the aim to increase FP use and access to reproductive health services for all women.

Modern contraceptive use in Niger is primarily characterized by pill and injectable use. In the 2012 Niger Demographic and Health Survey, among married modern FP method users, the pill was the most commonly used method (46%) followed by lactational amenorrhea method (LAM) at 32% and injectables at 17% ( Institut National de la Statistique (INS) et ICF International, 2013). By 2017, among married modern FP method users, the pill and injectable each represented about 40% of the method mix and implants increased to 17% ( PMA2020, 2018). Recent client exit interview data collected at 45 public-sector health centers in Dosso, Niger, show injectables as the most commonly used method, with 58% of clients surveyed using this method, followed by pills (28%) and the implant (13%) ( Speizer *et al*., 2021). This shift in hormonal method use from pills to injectables, and later to implant use, follows the same progression that has been detected in a number of countries in Sub-Saharan Africa ( Bertrand *et al*., 2020).

Many factors influence a woman’s FP method choice, including demand-side and supply-side factors. Studies from North America, Asia, and Sub-Saharan Africa have shown that women’s limited knowledge of fertility patterns and FP methods, conflicting norms and beliefs, fear of side effects, misconceptions about modern methods, number of living children, peers’ method use, and partner’s acceptability of the method impact method use and choice ( Adinma *et al*., 1998; Ajong *et al*., 2018; Brunie *et al*., 2019; Calhoun *et al*., 2022; Gueye *et al*., 2015; Higgins *et al*., 2020; Izale *et al*., 2014; Moronkola *et al*., 2006; Odwe *et al*., 2021; Sullivan *et al*., 2006; Valente *et al*., 1997; Wasti *et al*., 2017). The method’s effectiveness and the safety of the method in terms of a woman’s health have also been found to be influential in decisions around method choice ( Adinma *et al.*, 1998; Higgins *et al*., 2020; Moronkola *et al*., 2006). Structural and supply side barriers influence method choice through stock outs or limited supplies of FP products ( Zuniga *et al*., 2022), and provider bias ( Solo & Festin, 2019). In an analysis of quantitative and qualitative data from Burkina Faso and Uganda, Brunie & colleagues (2019) demonstrate through quantitative analyses that effectiveness, duration of use, side effects, cost, and access are important factors to women when choosing a FP method. Their qualitative data show that bleeding side effects, duration of use, discretion of the method, convenience of the method, predictability of side effects and cost were most important. The study by Brunie *et al*. (2019) demonstrates the added insights and additional themes that qualitative data can provide in examining what method attributes are important to consider for family planning programming.

FP providers play an integral role in supporting women’s access to an expanded method choice and are often considered trusted sources of knowledge for FP ( Gosavi *et al*., 2016; Higgins *et al*., 2016). A study in Niger using data collected in 2014 showed that 67% and 75% of women aged 15–19 and 20–24 years, respectively, prefer to receive information about FP methods from health centers ( GRADE Africa, 2021). Quality counseling on FP methods and their side effects at method initiation and additional counseling during the antenatal and postpartum period have been shown to improve continuation and increase perinatal contraceptive uptake respectively ( Cavallaro *et al*., 2020). Providers can also serve as barriers to clients’ access to a full range of methods. A study in urban Nigeria found that many providers restricted access to methods based on age and that other eligibility criteria, such as parity and marital status, were also imposed ( Schwandt *et al*., 2017). A review by Solo and Festin showed that providers’ bias towards clients based on age, marital status, or HIV status and their bias for or against specific methods influence the methods that clients are informed of and offered ( Solo & Festin, 2019).

Given providers’ crucial role in FP counseling and method provision, understanding their perspectives on method attributes and their clients’ method preferences is important to efforts to improve use of and access to a full range of methods. This study seeks to gain a better understanding of providers’ views on the suitability of different hormonal FP methods for women using qualitative data from in-depth interviews (IDIs) with FP providers. These data provide detailed information and nuanced insight into what attributes of FP methods providers feel are desirable for women in Niger.

This study has been reported in line with the Standards for Reporting Qualitative Research (SRQR) guidelines ( Speizer, 2022).

## Methods

### Study setting

The data for this study were collected as part of a larger assessment of a FP segmentation counseling tool used by FP providers with clients in government run integrated health centers (IHCs) in Niger. The study collected quantitative and qualitative data and was conducted at 45 IHCs in Boboye, Dosso, Doutchi, Falmey, Loga, and Tibiri health districts in Dosso region. Dosso is a region in the south western part of Niger, where the majority of the population live in a rural setting. The segmentation strategy involved providers asking clients a series of 12 questions and based on the responses of the clients, the providers segmented clients into one of five groups for targeted counseling.

### Study design

The overall assessment of the segmentation tool was designed with three study arms: Arm 1 was comprised of IHCs with a demand generation program and the segmentation strategy, Arm 2 included IHCs with the segmentation strategy, and Arm 3 served as a control arm where the IHCs did not have any specific demand generation or segmentation activities. Providers from all three arms (8 per arm) were interviewed about their experience providing FP services and in addition, providers in Arms 1 and 2 were asked about their experience with the segmentation counseling tool.

This qualitative study used a thematic analysis approach to analyze in-depth interview data. The semi-structured interview guide was designed with two parts. Part 1 included two vignettes of hypothetical FP clients: a 17 year old, unmarried, nulliparous adolescent who was seeking FP; and a 23 year old married woman with two children seeking a FP method. Each vignette was followed by questions about how the provider would navigate a consultation with the client and what FP methods they would recommend for each client. Part 2 of the interview guide included questions about providers’ experiences with and opinions about the segmentation tool (Arms 1 and 2 only). The interview guide was pilot tested with four providers working in IHCs outside the study area before data collection began. The guides were tested for clarity, flow, and to ensure the questions were appropriate. Modifications to the questions and the guide were made based on feedback from the pilot testing. The final guides used can be found as *Extended data* ( Speizer, 2021). The qualitative data used for the analyses described in this paper are comprised of Part 1 of the 24 in-depth interviews (IDIs) collected with providers across all three study arms. Information on provider perspectives on the segmentation tool (Arms 1 and 2) are provided elsewhere ( MacLachlan *et al*., 2022).

### Data collection

Data were collected in February and March of 2020. Two interviewers, one female and one male, conducted the 24 IDIs, with each interviewer responsible for 12 interviews. Interviewers, hired by GRADE Africa as part of the data collection team, were not age or gender matched with interviewees. All participants provided written informed consent prior to being interviewed. Interviews were conducted in a private room or space within or close to study IHC where each of the providers worked. The interviews were audio recorded with the written consent of participants. The interviews were conducted in French, Hausa, or Zarma, depending on the comfort level and preference of the interviewee. The duration of the interviews ranged from 33 minutes to 104 minutes.

### Analysis

All audio recordings of the IDIs were translated from Hausa and Zarma and transcribed into French by trained translators and transcriptionists in Niamey, Niger. During data collection, four transcriptions were compared to audio recordings by the supervisor in Niger to ensure the fidelity of the transcriptions. Once all interviews had been transcribed into French, de-identified and anonymized, transcripts were uploaded for analysis to Dedoose version 9.0.46, ^1^ a qualitative analysis software that permits collaboration ( Dedoose, 2021). A preliminary codebook was created by the master coder (EM) based on the interview guide questions and an initial review of three randomly selected interview transcripts and was entered into Dedoose for coding. The codebook included major thematic codes called “parent” codes and smaller sub-thematic codes referred to as “child” codes. This preliminary code book was used by the master coder to code six interviews. The six coded interviews were reviewed by all coders after which all coding and the code book were revised based on discussion among all coders. Four coders (AMJ, BA, EM, SC) then applied all the parent codes to the remaining interviews and two coders (AMJ, BA) then applied child codes. All four coders involved in coding parent codes had an average Cohen’s kappa score of 0.79 and a range of 0.72 to 0.89, when each was compared to a master coder ( Gwet, 2014; Landis & Koch, 1977). Thematic analysis was then completed and summarized for each family planning method discussed in the interviews. The quotes presented in this paper were translated from French into English by the first author and reviewed and approved by the Niger study team to ensure accurate interpretations.

### Ethical approval

Ethical approval for all consent procedures, surveys, and IDI guides was obtained from the National Ethics Committee for Health Research (CNERS) in Niger (#049/2019; approved 14 Jan. 2020), and the University of North Carolina at Chapel Hill’s Institutional Review Board (#19-3042; approved 3 Jan. 2020).

## Results

### Provider characteristics

One provider from each of the 24 IHCs selected for the study participated in the IDIs. Providers ranged in age from 25 years to 59 years, were predominantly female (83%), and had worked as FP providers for 1 to 28 years, with a mean of 7.5 years of experience (see Table 1). Providers interviewed included chief of IHC (12%), deputy chief of IHC (17%), midwife (25%), nurse (21%), FP provider (17%), and volunteer (8%).

**Table 1.** Provider characteristics.

Total providers interviewed	24
Female providers	20
Male providers	4
Provider age (years)	
25–29	4
30–34	10
35–39	6
40–44	0
45–49	1
50–54	1
55+	2
Years working in family planning	
1–4 years	11
5–9 years	5
10–14 years	5
15–19 years	2
20+ years	1
Type of family planning provider	
Chief of integrated health center	3
Deputy chief of integrated health center	4
Midwife	6
Nurse	5
Family planning provider	4
Volunteer	2

### Summary of main findings

The overwhelming majority of providers (92%) stated that a provider’s role is not to recommend a contraceptive method and that it is up to the client and in some cases her husband, to decide which method to use. When probed further by the interviewer and asked which method they believed would be most suitable in each of the vignettes, most providers named the contraceptive implant to be the most suitable, for both the married and unmarried client. Regarding women’s method preferences (according to providers) one of the most significant themes emerging from the interviews was that the injectable is the most popular FP method for women coming to the IHC. Relatedly, when asked what method they thought each hypothetical client would choose, the majority of providers said that women overwhelmingly would prefer injectables. However, providers stated that while some women may not initially choose the implant, once they were told more about it, they were more open to adopting it and less likely to believe negative local myths about implants. Overall, providers did not consider the contraceptive pill to be desirable by women due to its lack of discretion and challenges associated with effective compliance.

In content analysis of interview data about the characteristics and attributes of the FP methods available and chosen by clients, several key themes emerged. The themes identified were: (a) the discretion of the method; (b) the simplicity of the use of the method; (c) comfort and familiarity with the method; (d) myths and misconceptions about methods; (e) husband opposition to the implant; and (f) concerns about return to fertility post method use. The results are presented below by the main themes that emerged through the analysis and a summary of these results can be seen in in Table 2.

**Table 2.** Summary of results by main theme.

Main themes from the analysis	Results
Discretion of method	Pills are the least discreet on daily basis Pills disrupt women’s menstrual cycles the least Providers divided about whether the injectable is more discreet than the implant.
Simplicity of use of a method	Implant limits risk of non-compliance Implant requires no refill or re-injection visits
Comfort and familiarity with the family planning method	Pills and injectables have longer history in Niger Women come to family planning visit with preference for injectables based on recommendations from others
Myths and misconceptions about methods	Women fear: - Going to hell if they die with an implant in their arm - Implant getting lost in their body
Husband opposition to the implant	Husbands can demand implant removal Implant use can cause of marital problems
Concerns about return to fertility post method use	Implants offer immediate return to fertility after removal Injectables not recommended for adolescents due to concerns of delayed return to fertility

### Discretion of method

The providers felt that many women want a FP method that is discreet— providers stated that the majority of women seen at a IHC want to keep FP use a secret from their parents, their sexual partners, their husbands and the community at large. When asked about what method women prefer, providers would compare the methods in terms of discretion, such as this comparison of injectables and the implant versus oral contraceptive pills for adolescents:

“Interviewer: Except for forgetting, for what other reasons do you think that pills are less suitable for her?
Provider: They are less suitable because she is unmarried, once she arrives at home, you can see her with the pills and it’s a whole problem for her, but if it is injectables or the implant no one can see it.”
-    Female provider, Age 35

Many providers expressed their opinion that pills are the least discreet method choice for women as they are taken daily and can be found in their belongings. One provider described the inevitability of pills being discovered in this comparison of the implant and pills:

“Interviewer: What are the reasons that would motivate the choice of the implant?
Provider: Because it is discreet, no one can know that she uses contraception, whereas if it is the pill, sooner or later someone will see it.”
-    Female provider, Age 35

However, in one regard, pills were considered discreet. According to providers, pills were the method that disrupted women’s menstrual cycles the least when compared to injectables and the implant. This benefit relates to young women who want to keep their FP use secret and whose parents or sexual partners may notice changes in their menstrual cycle caused by their FP use. In contrast, injectable and implant use may be discovered as they are reported to cause more disruptions in menstruation and irregular bleeding. 

Providers had divided thoughts about whether the injectable is more discreet than the implant. For almost all providers, the discreet nature of the implant was an important factor in naming the implant as the most suitable method for women. Some called the implant “invisible” or “secret” and stated that no one, even a woman’s husband, will know that she has an implant. Furthermore, providers reported that women share these same beliefs about the implant.

“…But they themselves, they prefer the implants in the sense that after having inserted it, no one knows that they are wearing it. But if they use injectables or pills, it is possible that someone knows the situation that they are in. Like, understand what they are doing. But as soon as they use implants, no one knows the situation in which the girl finds herself, unless she reveals her secret herself.”
-    Female provider, Age 31

For other providers the visibility of the implant itself in the arm and/or the bandage following insertion is a downside to the implant since both can be seen by others. These providers stated they would even advise against an implant for a woman since people will know she is using FP. For providers who think that injectables are more discreet, they often mentioned that the injection cannot be seen like the scars left over from implant insertion:

“Yes because injectables if she does it it’s an injection. When she does that it’s done because no one can discover she has used injectables. Whereas the implant it’s the type that is under the skin. And maybe accidentally someone can discover that she has [it], and someone that knows what it is, can discover in seeing her arm, what does she have under her arm? And that people can discover that there you go, she has an implant. So that’s it and there are always suppositions...”
-    Male provider, Age 39

Providers also report that women voice the sentiment that injectables are less conspicuous than implants to them:

“Well you know some say that if they take the implant, you can see the scars on their arms, that is why they do not like taking it. It is injectables that the majority of clients choose because, in their opinion, it is a discreet method that no one will know she has taken.”
-    Female provider, Age 30

However, some providers felt that even though the injection is discreet, it is possible injectable users can be discovered because they have to go to the clinic every three months for reinjection and cannot explain these IHC visits to their husbands and others. Providers shared that both married and unmarried women prefer not to be seen at the health center for fear of what others may think of them. This is especially true for adolescents, who will even come to the clinic at night for their follow-up injections to avoid detection:

“Interviewer: What about young clients who come for FP, you said earlier, you are from the village, some avoid you, do they go to the other providers?
Provider: Now, I really do not know, but before, there were young adolescents who came at night to get injectables.”
-    Female provider, Age 35

In contrast, the implant provides discretion for women by reducing IHC visits to a minimum.

“I propose the implant because not only would I say it is discreet, but it is also reliable, her husband might also never discover that she is using FP because she does not come every month or every three months to the IHC for FP, her husband will suspect that is what she is going to do whereas it is just one time with the implant, she comes and it is finished.
-    Male provider, Age 39

The level of discretion a method can provide is a decisive factor that providers feel affects the preferences of clients and affects their method choice. While differing opinions of the providers are reported regarding whether or not the implant or injectable is more discreet, the discretion of the implant and injectables is an important factor for providers and their clients.

### Simplicity of use of a method

Overall, providers expressed widespread support for the use of implants by both married and unmarried women due to their effectiveness, long-acting nature, and the simplicity of their use. A principal reason providers favor implants is that there is no risk of non-compliance with using this method of FP. Once inserted into a woman’s arm, she does not need to take a daily dose or return to the health center for refills or re-injections. Providers frequently contrasted this characteristic of the implant with the risk of clients forgetting to take the pill correctly or not returning for injections every three months, thus putting themselves at risk for pregnancy. Many providers strongly recommend the implant for adolescent users, who they believed would struggle with forgetfulness, for these reasons.

“In my opinion, it is the implant that would suit them. If I clearly explain to her the different methods, she will understand that there are methods that once applied there is a determined period after which you remove them. The other methods make it so women will regularly go to the infirmary. And these methods are characterized by forgetting or by errors in compliance. You can also forget the appointments.
-    Female provider, Age 30

Other providers stated that married women with children may be more forgetful than younger women since they have more responsibilities to manage. Similarly, some providers put women’s preference for the 3 month injectable schedule in contrast to the daily use of oral contraceptive pills. These providers indicated that the relatively long time period of three months between injections was appreciated by the women, who could put off going to the IHC at least during those months:

“ ‘Sayana’ or injectables are taken every three months as opposed to pills, it is every day and the woman must take them at the same time. If she forgets to take the pill one time, she can become pregnant. On the other hand, ‘Sayana’, the injectable is done at the IHC and she only renews it three months later at an IHC.”
-    Female provider, Age 35

### Comfort and familiarity with the family planning method

Another theme that emerged from the interviews was the idea of women’s familiarity and overall comfort, both physical and psychological, with the various FP methods. Pills and injectables have a longer history in Niger. One provider eloquently explained the historic popularity of injectables as one of the only FP methods available to women outside of oral contraceptive pills:

“Interviewer: For the woman that we just described [an unmarried and nulliparous 17 year old], I’d like to know if she physically presented herself in front of you and after having explained the different methods available, which method do you think she will choose? Because after the explanation you have an idea of what method she will choose.
Provider: It’s the injectable that she will choose, like I just explained to you.
Interviewer: Why?
Provider: Ahh, it is their mentality. They only prefer injectables. It’s now with the evolution of the change in methods. There are others, you will do everything, the explanation, physical presentation of the briefcase [of methods], they will say that I want the injectable.
Interviewer: Why do you think they have this idea of wanting injectables?
Provider: Simply because in the past when we provided FP services, it was only injectables and pills. And if they take pills, they easily forget, whereas with injectables they know that it’s for three months, so they cannot forget. In other words, in years past there was not the implant, it was only the pill and the injectable.”
-    Female provider, Age 59

Many providers described this preference as starting in the community during discussions between women; a large proportion of whom are already injectable users encourage other women who need FP to come to the IHC to start injectables. Women can sometimes be reluctant to try any other method, even with counseling:

“Provider: It’s the conversations, if a woman comes, she does not let someone else come and we present them different methods, she will say that me, I got injectables and since it did not cause any side effects, she will tell her friend if you go you have to take this too, they already have this in mind when they come here. They say I want the injectable.
Interviewer: So what do you think, does this mean that friend’s and acquaintance’s choice have a bigger influence on method choice than what you propose as a health care provider?
Provider: That’s it. Since even if you try to explain, like I just said, if in my opinion I will give her the implant, but if they come they already have a method in mind, whatever efforts you make to explain here are the side effects, she will tell you yes, but this is what I want. That is what they say.”
-    Female provider, Age 28

Even so, a strong theme that emerged was that women will come into the clinic with injectables as their first choice but will then switch to implants after counseling from the provider. Providers shared that clients, both new and returning, when “counselled well” and presented with the full range of methods, will sometimes switch their choice to the implant. This occurrence happens at IHCs because when informed about the implant, women appreciate the implant’s attributes when compared to injectables:

“Sometimes a woman presents herself and says that she has come to get injectables. But when you present and explain the briefcase of methods, they prefer the implant. Those who chose pills are the exceptions.”
-    Female provider, Age 30

Women are also hesitant to choose implants because of their fear of the pain of implant insertion and removal. Providers mentioned several times that women, after hearing stories in their communities about the process of insertion and removal, fear the implant.

“Or someone said I had a wound on my arm when they gave me the implant, the others will say that can happen to them if they get the implant. Others say that in getting the implant, they cut your arm to put it in and at the moment of removal they have to cut into you to remove it, so fear will make it so they refuse to change methods. Sometimes really it is these rumors from others that frighten women.”
-     Female provider, Age 32

Providers recounted that clients have heard negative stories of women’s experiences of pain due to implant insertion and/or removal, perhaps without receiving anesthesia. The providers gave examples of women who initially refuse the implant due to the fear of being cut and then accept it after hearing an explanation of the process and use of anesthesia.

“Provider: Before clients had prejudice towards implants? They said to themselves that the insertion was painful. The local name for the implant was “tear.” But now with information campaigns they accept the implant.
Interviewer: How have you increased their awareness?
Provider: We explain the insertion procedures to them. It suffices to numb the part, and the implants are inserted even without the client noticing it.”
-    Female provider, Age 30

### Myths and misconceptions about methods

Out of all the methods, the most discussion of rumors was surrounding implants. Several rumors in the community were mentioned by providers, including beliefs that a woman who dies with an implant in her arm will go to hell or not be able to “reach paradise”, and that if a woman gains too much weight with an implant, the implant can get lost in her body.

“I believe that they are more comfortable with injectables. But she rejects the implant just because of religious reasons which say that if you die with an implant in your arm you go directly to hell. There is also the thought that if a woman gains a bit of weight the implants disappear in her body. We always try to provide information. Some understand, others do not.”
-    Female provider, Age 30

These myths and misconceptions are described as having a powerful influence over women’s contraceptive method choice and they counter providers’ appreciation for the implant as a method. Providers discussed having to address these perceptions among women through counseling or by encouraging satisfied implant users to help dispel implant myths in their communities. While providers state that these myths are common, some providers shared that women’s attitudes and openness towards implants are changing with more education and counseling about FP methods.

“I told them you also, you have to go tell people what you have seen, and also people should no longer say that when you get an implant, if you die, you will go directly to hell, so you should not believe in that.”
-    Female provider, Age 33

### Husband opposition to the implant

While providers spoke of husbands being opposed to FP use generally, some providers also discussed husbands specifically being opposed to their wives using an implant as a method of FP. They mentioned that a woman’s use of an implant could be contentious enough as to cause serious marital problems between her and her husband. Four providers gave specific examples of instances when a client returned to the health center and demanded that the implant be removed at the request of her husband. In the excerpt below, one provider recounts a time when a client returned with her husband who demanded she remove her implant but accepted her use of injectables instead.

“Interviewer: Have you had cases where the husband comes to complain about the contraception that his wife is using?
Provider: At my level of service, I have not encountered cases of complaints. But in the village, I hear of rumors where the woman is even threatened at home. But it is true I had a case where the husband threatened to kick his wife out if she did not remove the implant that she had inserted.
Interviewer: Did you remove it for her?
Provider: I asked that the woman bring her husband. I counseled them, after which the husband understood the importance of FP. Instead of stopping, he asked to change method. He preferred to remove the implant and take injectables. He made a change in method.”
-    Male provider, Age 26

### Concerns about return to fertility post method use

One reason providers preferred implants for both married and unmarried women is the rapid return to fertility after removal of the implant relative to injectables or pills. Providers stated that once a woman decides she wants to become pregnant, she can remove the implant and conceive a child without delay. The immediate return of fertility is particularly important for unmarried clients who could potentially get married at any point while using the implant and want to start childbearing soon after marriage.

“Interviewer: What other information can you share with her on these long-acting methods that she has chosen?
Provider: On the chosen method? You see I will tell them that the implant here that she has already chosen is an efficient method and it is a method that when she will have the chance to get married and want to get pregnant if she removes it, she will have a pregnancy without problem.”
-    Female provider, Age 31

Providers felt strongly about injectables as the wrong choice when compared to the implant when serving adolescents. Providers’ preference for the implant for adolescents is mainly due to injectables’ known side effect of delaying the return to fertility after stopping use ( Barden-O’Fallon *et al*., 2021) and the implications this has for adolescents wanting to become pregnant when they marry later on:

“Interviewer: If you still keep in mind her age of 17 years, unmarried, without children, has never used FP, and does not want children in the next two years, in the logic of this example, which methods will you choose for this client?
Provider: The implant
Interviewer: What other methods?
Provider: Implants, Implanon or jadelle
Interviewer: Why these methods?
Provider: Because for these methods the return to fecundability does not take time. As soon as she removes it she can get pregnant. Whereas injectables bring a delay that brings women on the quest for sterility. So for a client who has never given birth the preference is for her to use an implant.”
-    Female provider, Age 30

At least one provider mentioned that the delay in return to fertility, that is a common side of effect of stopping injectables, can lead to permanent sterility:

“Because you know all the contraceptives are used according to their duration of action. After one it is the other. After the progesterone only pill it is the combined oral contraceptive pill that comes after six months of breastfeeding. Usually injectables are indicated for a women who has at least three children. But now in the health system there are a lot of things that happen just like that. Because there are women when they take injectables they can no longer get pregnant.”
-    Male provider, Age 36

As previously discussed, changes to bleeding and menstruation cycles were a side effect that providers stated some clients found to be troublesome and undesirable especially if they make the method more detectable; however, this did not emerge as a major theme beyond how they related to return to fecundability.

## Discussion

Providers play an integral role in clients’ method selection as well as having access to privileged insight into the reasons behind client’s method choice. This qualitative study found that providers felt that implants were the most suitable method for the 17-year old unmarried woman without children and for the 23-year old married woman with 2 children described in the vignettes; however, they also believed that both women would choose injectables instead. The level of discretion that a method offers to women emerged as the most prominent theme that providers felt was important to women when considering which method was most suitable for them. Another important consideration was simplicity of use and ease of adherence to the method and their implications on a method’s effectiveness. Providers reported that women’s familiarity with injectables, other women’s method recommendations, myths and misconceptions within the community about implants, and husband’s disapproval of implants influenced women’s FP method choices and contributed to women’s preference for injectables. The delay in the return to fertility was a side effect that providers believed to be important when considering which method would be the best fit for a woman and was an often-cited reason for believing the implant was the most suitable for a woman, regardless of marital status.

These themes appear in other studies on FP method preferences, but many of the studies use quantitative methods. The IDIs with FP providers used for this study provide nuanced insight into their views on the attributes of the implant, injectables, and pills and the contraceptive needs and preferences of their clients. In a mixed-methods study that was conducted in Burkina Faso and Uganda in 2016–2017 to investigate preferred method characteristics from women, men, and providers, quantitative results showed that method effectiveness, duration of contraceptive coverage, side effects, cost, and access were the characteristics most reported as important by women in both countries ( Brunie *et al*., 2019). In Burkina Faso, the quantitative data illustrated that discreet use of the method was an additional desirable characteristic, but this was not reported as frequently as the other characteristics listed above. Conversely, in qualitative data, discreet use and side effects emerged as the highest-ranking method characteristics reported by women through focus group discussions and providers through IDIs in both countries. Other characteristics that the qualitative data showed as important included the quick return to fertility, partner approval of the method, and family or friends recommending the method ( Brunie *et al*., 2019). This aligned with what was found in our data suggesting that qualitative studies identify different valued features of methods than quantitative studies.

In a predominately Muslim society like Niger where early marriage is common and family planning is not normative ( Samandari *et al*., 2019), FP use is something women would like to keep private ( Baiden *et al*., 2016; Silverman *et al*., 2020) and therefore how discreet a method is plays an important role in method preferences for women. Providers in our study voiced discrepant perspectives regarding whether implants or injectables were the more discreet method. Some providers felt that the implant was visible to others while others felt it would not be seen. Further, some providers felt that injectables, that require visits to the health facility every three months, may be less discreet than the implant that requires less frequent visits.

Women’s preference for injectables, as reported by providers, was mainly related to the belief that they are the most discreet method and to women’s familiarity with this method. Pills have been a commonly used modern method of FP in Niger; a provider explained that pills and injectables have a longer history in Niger than other FP methods like implants. In a sample of clients from health centers in Dosso, Niger, injectables were the most commonly used method, confirming the reports from providers that clients prefer injectables ( Speizer *et al*., 2021). Notably, providers did not think that pills fulfilled the needs of women very well. Pills were considered to be the least discreet method and were also regarded as difficult for women to remember to take properly.

While injectables are the most common method chosen by women in Dosso, a main theme that came out of the IDIs was that when clients were counseled on all methods, a number of them would identify that the implant fit their FP needs the best. Since 2014, implant use has rapidly and considerably increased throughout Sub-Saharan Africa including Niger ( Jacobstein, 2018). This can be attributed to the method’s positive attributes, updated eligibility guidance, increased availability, and lower commodity costs ( Jacobstein, 2018). The transition in hormonal method mix from pills, to injectables, to implants is a documented trend in a number of places in Sub-Saharan Africa ( Bertrand *et al*., 2020).

Important barriers to implant use discussed by providers were myths and misperceptions about implants and partner disapproval of implants. A study in Ethiopia found that 67% of women surveyed had heard myths and misconceptions about long acting and permanent methods, including implants ( Meskele & Mekonnen, 2014). Providers in our study frequently discussed the commonly held misconception that a woman will go to hell if she dies with an implant in her arm. This misconception along with others were thought to discourage women from adopting the implant.

Partner approval of a FP method is associated with method choice ( Odwe *et al*., 2021) and husband disapproval of implants was discussed by providers as a reason for women not choosing or discontinuing implant use. Husband disapproval of implants was described as strong enough to create marital troubles for implant users whose husbands did not consent to their use. The findings of this study suggest that increasing awareness within the community of implants and unbiased counseling on all FP methods can be important to help ensure that women in Niger have access to an expanded method choice.

International aid funders, such as the Bill & Melinda Gates Foundation, have prioritized and made investments into developing new contraceptive technologies in order to better address women’s reproductive needs. Studies have been done in various countries in Sub-Saharan Africa to explore women’s openness and opinions about different characteristics of family planning methods ( Brunie *et al*, 2021; Callahan *et al*., 2019; Callahan *et al*., 2021; Cartwright *et al*., 2020). Similar to our study, one study found that duration of use, and familiarity with methods were important attributes of methods in Burkina Faso and Uganda; however, irregular bleeding emerged as a more important attribute to consider than in our study and discreet use was not as prominently discussed ( Callahan *et al*., 2019). A deeper understanding of women’s preferences and the degree of importance which various attributes of contraceptive methods hold for women can provide useful insight into family planning programs and the development of new contraceptive technologies.

This study has several limitations. First, this study uses data from providers only and does not include information from clients. Providers speak about the method they believe to be most suitable for women as well as their experience with what their clients have chosen. However, to get a better understanding of the reasons behind actual contraceptive choices, interviews with clients would be necessary. Second, for this analysis, we only undertook IDIs with 24 providers; a larger sample could have provided more in-depth information. Relatedly, while data were collected from three study arms, there were not enough observations in each arm to determine if responses to the scenarios differed by whether the provider was trained on the segmentation strategy. Third, the data from this study are from one region in Niger and are not representative of other regions of Niger or elsewhere in West Africa or beyond. 

## Conclusion

It is important to consider the characteristics of FP methods that matter the most to clients as funding goes into programs to promote FP use and to develop new contraceptive technologies. Furthermore, FP visits and counseling sessions are important opportunities for providers to not only provide FP methods but also to help women identify the method that best meets their needs as well as to allay concerns about method side effects to help ensure that all women are able to choose from a full range of methods when or if they want to use contraception. 

## Data availability

### Underlying data

The qualitative data generated and analyzed during the current study are not publicly available in order to protect the identities of the participants involved but are available from the last author ( speizer@email.unc.edu) on reasonable request that clarifies how the data will be used and provides plans for safeguarding the data in a manner that protects the participants identities.

### Extended data

UNC Dataverse: Full Access, Full Choice: Increasing Youth’s Access to Expanded Method Choice. https://doi.org/10.15139/S3/5OBCHL ( Speizer, 2021).

This project contains the following extended data:

- FAFC Niger Segmentation Study In-Depth Interview Guides

### Reporting guidelines

UNC Dataverse: Standards for Reporting Qualitative Research (SRQR) checklist for ‘Providers’ views on hormonal family planning methods: a qualitative study from Dosso, Niger’. https://doi.org/10.15139/S3/94QIKN ( Speizer, 2022).

Data are available under the terms of the Creative Commons Zero "No rights reserved" data waiver (CC0 1.0 Public domain dedication).

## Footnotes

^1^ For those seeking to use a similar qualitative data analysis software, Taguette is a free and open source qualitative data analysis software that has similar capabilities to the one used to undertake the analyses in this paper.
