# Supplementary material for: Providers’ views on hormonal family planning methods for young women: a qualitative study from Dosso, Niger
Source: Gates Open Res. 2022 Oct 3;6:75. Originally published 2022 Jul 4. [Version 2] doi: 10.12688/gatesopenres.13674.2 (PMC9550940; doi:10.12688/gatesopenres.13674.2)
Supplement: Peer review history

# Peer review: Reviewer response for version 2

**DOI:** 10.21956/gatesopenres.15337.r32628

**Published:** 2023-01-09

**Authors:** Dieudonne Bidashimwa

**Affiliations:** 1 FHI 360, Morrisville, NC, USA

The authors have adequately addressed all my comments.

Is the work clearly and accurately presented and does it cite the current literature?

Yes

If applicable, is the statistical analysis and its interpretation appropriate?

Not applicable

Are all the source data underlying the results available to ensure full reproducibility?

Yes

Is the study design appropriate and is the work technically sound?

Yes

Are the conclusions drawn adequately supported by the results?

Yes

Are sufficient details of methods and analysis provided to allow replication by others?

Partly

Reviewer Expertise:

Family planning and reproductive health, women's health, infectious diseases, and health policy

I confirm that I have read this submission and believe that I have an appropriate level of expertise to confirm that it is of an acceptable scientific standard.

# Peer review: Reviewer response for version 2

**DOI:** 10.21956/gatesopenres.15337.r32627

**Published:** 2022-10-10

**Authors:** Elizabeth E. Tolley

**Affiliations:** 1 Behavioral, Epidemiological & Clinical Sciences, FHI 360, Durham, NC, USA; 2 Maternal and Child Health Department, Gillings School of Public Health, UNC-Chapel Hill, Chapel Hill, NC, USA

This revised version, which includes a more detailed explanation of the segmentation study from which data were drawn, is much improved. I do not have any further comments or suggested revisions. From my perspective, I recommend accepting with no additional revisions.

Is the work clearly and accurately presented and does it cite the current literature?

Yes

If applicable, is the statistical analysis and its interpretation appropriate?

Not applicable

Are all the source data underlying the results available to ensure full reproducibility?

Yes

Is the study design appropriate and is the work technically sound?

Partly

Are the conclusions drawn adequately supported by the results?

Partly

Are sufficient details of methods and analysis provided to allow replication by others?

Partly

Reviewer Expertise:

I am a social science researcher with expertise in qualitative and mixed method research, with a focus on acceptability, preference, adherence and use of sexual and reproductive health technologies.

I confirm that I have read this submission and believe that I have an appropriate level of expertise to confirm that it is of an acceptable scientific standard.

# Peer review: Reviewer response for version 1

**DOI:** 10.21956/gatesopenres.14959.r32333

**Published:** 2022-08-23

**Authors:** Dieudonne Bidashimwa

**Affiliations:** 1 FHI 360, Morrisville, NC, USA

This article reports on FP providers’ perspectives on women’s choices and preferences of hormonal FP methods, and underlying reasons, in Niger. The results show that providers believe that both married and unmarried women might choose to use the implant and the injectable compared to other hormonal methods. However, there is no consensus on which of the two methods, implant or injectable, would be the most preferred. Providers also believe that women’s choice of contraception is shaped by a set of methods’ attributes and factors, the ability to conceal the method’s use, the method’s simplicity of use, comfort and familiarity with the method, myths and misconceptions around the method, husband opposition, and concerns about return to fertility after the method is discontinued.

 Overall, the article is scientifically sound. The authors did a good job applying an appropriate methodology for collecting, coding, and analyzing qualitative data. Particularly, data collection and analysis procedures are well described. The prominent findings are clearly and concisely explained. Results are summarized, discussed against the current knowledge, and some programmatic implications are suggested in the discussion and conclusion sections.

 However, the authors need to address three major problems in the manuscript. The major challenges are as follows:

1. The manuscript does not provide sufficient information on their sampling approach. Specifically, it is not clear how the 45IHC and the FP providers within each IHC were selected. More information is needed on the sampling design, inclusion criteria, and any challenges encountered during the selection of participants.
2. It would be helpful for readers, especially those without a strong background in FP, to understand the constructs measured by the two vignettes and their relevance for FP access or service provision. Also, please provide more details about the process of developing the vignettes (based on the literature or other sources? Which ones?) in the methods section.
3. Please, discuss more policy, programmatic, and research implications of your findings for the local and the global contexts.

The minor issues to address are listed below by section:

**Methods:**

**Setting**

- Please, provide more contextually relevant information about the Dosso regions, such as the mCPR/unmet needs or access to FP services.
- Also, explain more why the Dosso region was selected for the main project (FP segmentation)
**Study design**
- The second paragraph starts with some elements of the analytical process. For better clarity, use this paragraph only to describe the tools, and keep any details about the analytical approach in a separate paragraph.
- Provide details on how the two vignettes in part 1 of the questionnaire were developed and what they intended to measure.
- Provide more details about the questions asked as follow-ups to each vignette. For example, what do you mean by “how the provider would navigate the counseling?” Which aspects of the counseling did you ask the providers about?
**Analysis**
- Overall, the methodology for the analysis is very well described.
- Please, provide more information on how the apriori codes were developed (from the questionnaire, the literature, a conceptual framework, etc.?)
**Other comments on the methods**
- How did the study sample the 45 IHCs? What were the inclusion criteria?
- Provide details on the sampling approach for providers within each IHC. Did you select all the providers or one provider per IHC? If one provider was selected in each IHC, what criteria did you use to decide between two or more providers?

** Results**

**Provider characteristics**

- Remove the first sentence in this section and move it under a paragraph describing your sampling methodology in the methods section
- Are the chief and deputy chief of IHC and volunteers involved in the routine provision of FP services? If not, please discuss any implications for data validity in the limitations
**Summary of the findings**
- Please, differences between male and female providers if any
   
**Discretion**
- The last paragraph in this section is a bit repetitive. Please, revise to keep it concise.
**Simplicity of use**
- This section is mostly about method compliance than the simplicity of use per se. Consider changing the subtitle for more clarity.
**Comfort and familiarity with the family planning method**
- Please, clarify the highlighted section in the following sentence: “Many providers described this preference as starting in the community during discussions between women; a large proportion of whom are already injectable users encourage other women who need FP to come to the IHC to start injectables.”
- At first, it seems as if counseling has no effect in helping women decide to use another method than the injectable if they came to the IHC with clear recommendations from their friends or family members. Yet, the authors later mention that counseling could sway women’s choice towards the implant, even if they have a pre-determined choice for the injectable. This is referred to as a “strong theme.” Perhaps the authors should nuance between the two themes to clarify which of the two themes was more prominent.
**Concerns about return to fertility post method use**
- The first two sentences of the last quote are not very clear.

** Discussion**

- About FP misconceptions (see sentence: “This misconception along with others were thought to discourage women from adopting the implant.”) The sentence is true, but could you say more about the high prevalence and persistence of these misconceptions in Niger and other settings? For example, could this phenomenon be linked to a misalignment of FP SBCC with local cultural and religious considerations? Are there other factors explaining the misconceptions?
  ​​​​​​​
- About partners’ opposition to implants (see sentence: “The findings of this study also suggest that increasing awareness within the community of implants and unbiased counselling on all FP methods can be important to help ensure that women in Niger have access to an expanded method choice.”). From a social/gender norms perspective, this finding confirms the prevalence of gender norms and their impact on FP choice and use in Niger as reported in previous studies. This seems to indicate a need for (more) interventions and policies allowing women’s empowerment in making FP decisions themselves or jointly with their partners.

Is the work clearly and accurately presented and does it cite the current literature?

Yes

If applicable, is the statistical analysis and its interpretation appropriate?

Not applicable

Are all the source data underlying the results available to ensure full reproducibility?

Yes

Is the study design appropriate and is the work technically sound?

Yes

Are the conclusions drawn adequately supported by the results?

Yes

Are sufficient details of methods and analysis provided to allow replication by others?

Partly

Reviewer Expertise:

Family planning and reproductive health, women's health, infectious diseases, and health policy

I confirm that I have read this submission and believe that I have an appropriate level of expertise to confirm that it is of an acceptable scientific standard, however I have significant reservations, as outlined above.

# Response

**Published:** 2022-09-24

**Authors:** Ilene S. Speizer

**Affiliations:** University of North Carolina at Chapel Hill, USA

**Reviewer 3**

 Reviewer: Overall, the article is scientifically sound. The authors did a good job applying an appropriate methodology for collecting, coding, and analyzing qualitative data. Particularly, data collection and analysis procedures are well described. The prominent findings are clearly and concisely explained. Results are summarized, discussed against the current knowledge, and some programmatic implications are suggested in the discussion and conclusion sections. However, the authors need to address three major problems in the manuscript.

*Response: Thank you for the positive feedback. Responses to the three major areas are described following the description of those areas below.*

 Reviewer: The manuscript does not provide sufficient information on their sampling approach. Specifically, it is not clear how the 45 IHC and the FP providers within each IHC were selected. More information is needed on the sampling design, inclusion criteria, and any challenges encountered during the selection of participants.

*Response: This information has been added to the methods section.*

 Reviewer: It would be helpful for readers, especially those without a strong background in FP, to understand the constructs measured by the two vignettes and their relevance for FP access or service provision. Also, please provide more details about the process of developing the vignettes (based on the literature or other sources? Which ones?) in the methods section.

*Response: Based on this reviewer and another reviewer’s inputs, we have added greater detail into the methods section about the design and use of the vignettes.*

 Reviewer: Please, discuss more policy, programmatic, and research implications of your findings for the local and the global contexts.

*Response: This information has been added to the Discussion section of the paper.*

 Reviewer Minor issues, with responses below each one:

 Methods:

 Setting:

1. Please, provide more contextually relevant information about the Dosso regions, such as the mCPR/unmet needs or access to FP services.
2. Also, explain more why the Dosso region was selected for the main project (FP segmentation)

*Responses (Setting): Greater details on the setting have been incorporated into this section.*

 Study Design:

1. The second paragraph starts with some elements of the analytical process. For better clarity, use this paragraph only to describe the tools, and keep any details about the analytical approach in a separate paragraph.
2. Provide details on how the two vignettes in part 1 of the questionnaire were developed and what they intended to measure.
3. Provide more details about the questions asked as follow-ups to each vignette. For example, what do you mean by “how the provider would navigate the counseling?” Which aspects of the counseling did you ask the providers about?

*Response (Study Design): We have made the changes recommended including added details of probes and the design and focus of the vignettes, as requested in the overall comments by this reviewer.*

 Analysis:

1. Overall, the methodology for the analysis is very well described.
2. Please, provide more information on how the apriori codes were developed (from the questionnaire, the literature, a conceptual framework, etc.?)

*Response (Analysis): Thank you for the positive feedback on this section. We have added more information on development of the codes for analysis. A priori codes were developed from the interview guide and for the three hormonal methods of interest (implants, injectables, birth control pills). No other conceptual framework or literature source was used to develop our a priori codes.*

 Other comments on the methods:

1. How did the study sample the 45 IHCs? What were the inclusion criteria?
2. Provide details on the sampling approach for providers within each IHC. Did you select all the providers or one provider per IHC? If one provider was selected in each IHC, what criteria did you use to decide between two or more providers?

*Response: These additional details have been added, as requested in the overall comments above.*

 Reviewer (Results):

 Reviewer (Provider Characteristics):

1. Remove the first sentence in this section and move it under a paragraph describing your sampling methodology in the methods section.
2. Are the chief and deputy chief of IHC and volunteers involved in the routine provision of FP services? If not, please discuss any implications for data validity in the limitations.

*Response (Provider Characteristics): For the first item, this sentence has been moved. We have clarified in the text that all providers interviewed provided family planning services in their facilities as an eligibility criterion.*

 Reviewer (Summary of findings): Provide any differences between male and female providers if any

*Response (Summary): No differences were found by provider gender and this is now mentioned in the text based on this reviewer and reviewer 1’s inputs.*

 Reviewer (Discretion): The last paragraph in this section is a bit repetitive. Please, revise to keep it concise.

*Response (Discretion): This paragraph has been revised with this in mind.*

 Reviewer (Simplicity of Use): This section is mostly about method compliance than the simplicity of use per se. Consider changing the subtitle for more clarity.

*Response (Simplicity of Use): Thank you for pointing this out. We have changed this section to be Compliance with method use*

 Reviewer (Comfort and Familiarity with the FP Method):

1. Please, clarify the highlighted section in the following sentence: “Many providers described this preference as starting in the community during discussions between women; a large proportion of whom are already injectable users encourage other women who need FP to come to the IHC to start injectables.”
2. At first, it seems as if counseling has no effect in helping women decide to use another method than the injectable if they came to the IHC with clear recommendations from their friends or family members. Yet, the authors later mention that counseling could sway women’s choice towards the implant, even if they have a pre-determined choice for the injectable. This is referred to as a “strong theme.” Perhaps the authors should nuance between the two themes to clarify which of the two themes was more prominent.

*Response (Comfort and Familiarity with the FP Method): We have revised the complex sentence and have tried to better clarify the role of counseling and friend influences in this section. We have also clarified the importance of strong counseling in affecting women’s choices in contrast to their pre-determined preferences upon arrival at the facility.*

 Reviewer (Concerns about Return to Fertility Post Method Use): The first two sentences of the last quote are not clear.

*Response (Concerns about Return to Fertility Post Method Use): We have revised this quote to only include the important components that relate to this theme.*

 Reviewer:

 Discussion:

1. About FP misconceptions (see sentence: “This misconception along with others were thought to discourage women from adopting the implant.”) The sentence is true, but could you say more about the high prevalence and persistence of these misconceptions in Niger and other settings? For example, could this phenomenon be linked to a misalignment of FP SBCC with local cultural and religious considerations? Are there other factors explaining the misconceptions?
2. ​About partners’ opposition to implants (see sentence: “The findings of this study also suggest that increasing awareness within the community of implants and unbiased counselling on all FP methods can be important to help ensure that women in Niger have access to an expanded method choice.”). From a social/gender norms perspective, this finding confirms the prevalence of gender norms and their impact on FP choice and use in Niger as reported in previous studies. This seems to indicate a need for (more) interventions and policies allowing women’s empowerment in making FP decisions themselves or jointly with their partners.

*Response: Thank you for these inputs. Based on this reviewer’s and other reviewer’s comments, the discussion section has been revised to include greater details on issues raised by all reviewers. We agree with the reviewer that women need access to accurate and unbiased information about the implant and other methods in the community, not just from providers. Our study showed that they need this kind of education and information before they can truly be empowered to make FP decisions.*

# Peer review: Reviewer response for version 1

**DOI:** 10.21956/gatesopenres.14959.r32332

**Published:** 2022-08-22

**Authors:** Amanda Kalamar

**Affiliations:** 1 Population Council, Breakthrough RESEARCH, Washington, DC, USA

Overall, this is a well written and interesting paper, providing some much-needed nuanced qualitative data from FP providers in Niger. The paper is well motivated and referenced and the chosen quotes from providers in the results section are illuminating. Importantly, the results mainly focus on method attributes of hormonal family planning methods but there is no mention of how the vignettes, which focused on an adolescent and a young woman, were received by providers in a context where seeking FP services, especially by an unmarried adolescent, can be taboo. The discussion section could be strengthened by grappling with nuances and questions outlined below.

 Introduction:

- The objective of this manuscript and analysis includes the phrase “what attributes of FP method providers feel are desirable for women” – this can be influenced by a number of things such as social norms, their own biases and own experiences, and/or their understanding of their clients based on their experience as a provider. I’d like to see the authors acknowledge this in the discussion section and expand out a bit their discussion around the important role that providers play to include this.
- It’s not clear in the objective (or often in how the results are talked about) whether the focus is on adolescents and young women. I assume so given the ages of the clients in the vignettes, but it would be helpful to clarify and to be clear throughout what age groups these findings are focused on.

Methods:

- Can you say a little more about the providers – they work within an integrated health center but are they dedicated FP providers or do they provide a range of services? Do you have any information about how many clients, on average, they see and whether they see a lot of clients ages 15-24 (I’m trying to get a sense as to whether providers are responding more hypothetically to the vignettes about a 17-year-old adolescent and 23-year-old woman or more from experience with these particular age groups)?

Results:

- The authors find that “The overwhelming majority of providers (92%) stated that a provider’s role is not to recommend a contraceptive method and that it is up to the client and in some cases her husband, to decide which method to use.” – the nuance between *recommend* and *decide* seems important here. ‘Recommend’ is different from telling a client what to do and other studies have found that clients trust providers and want them to recommend a method and then the client can decide whether to take that recommendation. Did this nuance appear at all? Do the authors think that this is reflecting some social desirability bias (in this particular example or more generally)? I’d suggest that a discussion of this would be worthwhile to help a reader not over-interpret findings, as appropriate.
- The focus of the findings is mainly on the attributes of and comparisons between implants, injectables, and to some extent pills. But in Niger it’s very taboo to be an adolescent, especially an unmarried adolescent, seeking FP services – I’m surprised there aren’t more findings around the bias or stigma providers might have in these scenarios (though of course, this is hard to get at when interviewing providers themselves).
  - For example, the authors state: “Overall, providers expressed widespread support for the use of implants by both married and unmarried women due to their effectiveness, long-acting nature, and the simplicity of their use” – the widespread support is very surprising. Did this come up at all in the interviews? Either way, I would suggest the authors acknowledge this in the discussion section.
- Myths and misconceptions about methods: this section of findings is framed generally “about methods” but talks exclusively about the implant. Might this be hinting at too strong of a focus by providers on the implant? If the data exist, perhaps this section would be expanded to include other methods. If not, I would suggest that the authors reflect on this. A short discussion of this in the discussion section could be helpful.
  - I have similar questions about the exclusive focus on implants in the male opposition section.

Discussion:

- The inability to investigate whether these findings are consistent across providers in different arms is listed as a study limitation but one that likely needs more than a sentence in the limitation section. I would have liked to have seen some discussion around what the providers in Arms 1 and 2 are trained on as part of the study arm and whether findings should be interpreted with more caution than currently put forth: how much of the attributes of the implant that providers are citing as positive attributes come from their training and what they’ve been told about the method versus benefits about the method’s attributes that they’ve heard clients citing as reasons for selecting the method (especially those that came in for injectables initially)?
- I’d highlight caution of over-interpretation of the findings. The authors find that providers say the implant would be most suitable but there do seem to be mixed findings on which method provides the greatest amount of discretion and the authors note that in a setting such as Niger, discretion is often paramount. There also seem to be mixed findings in ‘simplicity of use a method’ as one of the two examples given highlights the preference for injectables over pills for this reason.
- While the authors do acknowledge that providers “believed that both women would choose injectables instead”, I’d love to see the discussion section grapple a bit more with this misalignment. Clients of course do change their minds during counseling if a method better aligns with their needs, but what might be some of the implications when providers think one method is most suitable for certain clients, but clients have a different preferred method (at least initially)?

Is the work clearly and accurately presented and does it cite the current literature?

Yes

If applicable, is the statistical analysis and its interpretation appropriate?

Not applicable

Are all the source data underlying the results available to ensure full reproducibility?

Yes

Is the study design appropriate and is the work technically sound?

Yes

Are the conclusions drawn adequately supported by the results?

Partly

Are sufficient details of methods and analysis provided to allow replication by others?

Partly

Reviewer Expertise:

Sexual and reproductive health research and program evaluation

I confirm that I have read this submission and believe that I have an appropriate level of expertise to confirm that it is of an acceptable scientific standard, however I have significant reservations, as outlined above.

# Response

**Published:** 2022-09-24

**Authors:** Ilene S. Speizer

**Affiliations:** University of North Carolina at Chapel Hill, USA

**Reviewer 2**

 Reviewer: Overall, this is a well written and interesting paper, providing some much-needed nuanced qualitative data from FP providers in Niger. The paper is well motivated and referenced and the chosen quotes from providers in the results section are illuminating. Importantly, the results mainly focus on method attributes of hormonal family planning methods but there is no mention of how the vignettes, which focused on an adolescent and a young woman, were received by providers in a context where seeking FP services, especially by an unmarried adolescent, can be taboo. The discussion section could be strengthened by grappling with nuances and questions outlined below.

*Response: Thank you for this positive feedback. We have added some detail into the methods section of the development of the vignettes. We included vignettes because we were seeking to understand how providers might respond to clients who would have important, but possibly less supported family planning needs.  Notably, many providers responded with a variety of different client scenarios and used our questions to summarize their thoughts in general about methods women may or may not like to use and why. As described in our response to Reviewer 1, providers did not say that the unmarried adolescent should not use FP. Any concern that providers expressed around unmarried FP users was that the hypothetical unmarried adolescent was sexually active, but that if she was, it was better for her to use FP for pregnancy prevention as well as for STI protection than not to.*

 Reviewer:

 Introduction:

- The objective of this manuscript and analysis includes the phrase “what attributes of FP method providers feel are desirable for women” – this can be influenced by a number of things such as social norms, their own biases and own experiences, and/or their understanding of their clients based on their experience as a provider. I’d like to see the authors acknowledge this in the discussion section and expand out a bit their discussion around the important role that providers play to include this.
- It’s not clear in the objective (or often in how the results are talked about) whether the focus is on adolescents and young women. I assume so given the ages of the clients in the vignettes, but it would be helpful to clarify and to be clear throughout what age groups these findings are focused on.

*Response: In the revised version of the manuscript we have edited the discussion section to address a number of Reviewer 2’s inputs, including addressing social barriers to family planning use that providers potentially impose based on their own comfort level and social norms. We have revised the introduction and objectives of the paper to clarify that providers are asked specifically about young women profiles but also provided input on use preferences for all women (see response regarding the use of vignettes). We have also changed the title so that it is clearer the focus on younger women.*

 Reviewer: Methods

- Can you say a little more about the providers – they work within an integrated health center but are they dedicated FP providers or do they provide a range of services? Do you have any information about how many clients, on average, they see and whether they see a lot of clients ages 15-24 (I’m trying to get a sense as to whether providers are responding more hypothetically to the vignettes about a 17-year-old adolescent and 23-year-old woman or more from experience with these particular age groups)?

*Response: All providers included in this study offered family planning services in their facilities, no matter their job title. This was a key selection priority of identifying eligible providers.  That said, providers also provided other health care services in the facility, given the size of the facilities and lack of other nearby facilities.  We have added to the text that all providers offered family planning services.  While we do not have routine data for these facilities, we have included information from the exit interviews in these same facilities to demonstrate that about 13% of family planning clients in the facilities were ages 15-19 years and 28% were ages 20-24 years; most of these clients were married and had two or more children. Thus, the hypothetical young clients are realistic; however, the unmarried nulliparous client is definitely an exceptional case.*

 Reviewer: Results

- The authors find that “The overwhelming majority of providers (92%) stated that a provider’s role is not to recommend a contraceptive method and that it is up to the client and in some cases her husband, to decide which method to use.” – the nuance between *recommend* and *decide* seems important here. ‘Recommend’ is different from telling a client what to do and other studies have found that clients trust providers and want them to recommend a method and then the client can decide whether to take that recommendation. Did this nuance appear at all? Do the authors think that this is reflecting some social desirability bias (in this particular example or more generally)? I’d suggest that a discussion of this would be worthwhile to help a reader not over-interpret findings, as appropriate.
- The focus of the findings is mainly on the attributes of and comparisons between implants, injectables, and to some extent pills. But in Niger it’s very taboo to be an adolescent, especially an unmarried adolescent, seeking FP services – I’m surprised there aren’t more findings around the bias or stigma providers might have in these scenarios (though of course, this is hard to get at when interviewing providers themselves).
- For example, the authors state: “Overall, providers expressed widespread support for the use of implants by both married and unmarried women due to their effectiveness, long-acting nature, and the simplicity of their use” – the widespread support is very surprising. Did this come up at all in the interviews? Either way, I would suggest the authors acknowledge this in the discussion section.
- Myths and misconceptions about methods: this section of findings is framed generally “about methods” but talks exclusively about the implant. Might this be hinting at too strong of a focus by providers on the implant? If the data exist, perhaps this section would be expanded to include other methods. If not, I would suggest that the authors reflect on this. A short discussion of this in the discussion section could be helpful.
  - I have similar questions about the exclusive focus on implants in the male opposition section.

*Response (Results): *

- *For the first bullet, we think this is a question of word choice. The providers felt passionately that it was not their choice, it was the client’s choice as to what method to adopt. We have retained recommend because we used this word choice in our study tools. Unfortunately we do not have enough additional data to make any more nuanced interpretations since the counseling process was not the main aim of this analysis and there were very few questions about this process in the interview guide. There certainly could be social desirability bias if these providers were trained in adolescent-friendly services and had been heavily trained in client choice. This possibility has been added to the Discussion.*
- *We have incorporated into the results a brief discussion on the providers’ perspectives on non-marital sex and contraceptive use that addresses this point. Basically, providers did not condone non-marital sex but also acknowledged that if the young person was having non-marital sex, better that she uses a method to avoid an unintended pregnancy or sexually transmitted infection.  This has also been added to the discussion. *
- *Although providers responded to hypothetical vignettes (i.e., scenarios) that involved unmarried adolescents, in reality the providers seemed to very rarely interact with unmarried adolescents due to the extremely strong stigma in Niger that the reviewer points out. This has been noted in the limitations section of the paper. *
- *We did find widespread support for use of implants among the providers in the study. That said, providers acknowledge that clients have different preferences and typically chose the injectable. This has been clarified in the text.  *
- *The reviewer is correct that the section on myths and misconceptions focuses on implants and the title of this section has been changed accordingly. *

Reviewer: Discussion:

- The inability to investigate whether these findings are consistent across providers in different arms is listed as a study limitation but one that likely needs more than a sentence in the limitation section. I would have liked to have seen some discussion around what the providers in Arms 1 and 2 are trained on as part of the study arm and whether findings should be interpreted with more caution than currently put forth: how much of the attributes of the implant that providers are citing as positive attributes come from their training and what they’ve been told about the method versus benefits about the method’s attributes that they’ve heard clients citing as reasons for selecting the method (especially those that came in for injectables initially)?
- I’d highlight caution of over-interpretation of the findings. The authors find that providers say the implant would be most suitable but there do seem to be mixed findings on which method provides the greatest amount of discretion and the authors note that in a setting such as Niger, discretion is often paramount. There also seem to be mixed findings in ‘simplicity of use a method’ as one of the two examples given highlights the preference for injectables over pills for this reason.
- While the authors do acknowledge that providers “believed that both women would choose injectables instead”, I’d love to see the discussion section grapple a bit more with this misalignment. Clients of course do change their minds during counseling if a method better aligns with their needs, but what might be some of the implications when providers think one method is most suitable for certain clients, but clients have a different preferred method (at least initially)?

*Response: We have revised the overview of the segmentation counseling strategy to provide more context on what the providers were trained on. In addition, based on this reviewer’s comments and those of the other reviewers, we have revised the discussion section to address many of the points raised by this reviewer, including over-interpretation of findings and discussion about the mismatch between provider and client re. method choice (especially the implant).*

# Peer review: Reviewer response for version 1

**DOI:** 10.21956/gatesopenres.14959.r32226

**Published:** 2022-07-25

**Authors:** Elizabeth E. Tolley

**Affiliations:** 1 Behavioral, Epidemiological & Clinical Sciences, FHI 360, Durham, NC, USA; 2 Maternal and Child Health Department, Gillings School of Public Health, UNC-Chapel Hill, Chapel Hill, NC, USA

Overall: This paper presents qualitative findings from a subset of Family Planning (FP) providers in Niger who took part in a larger study to assess the effects of a FP segmentation counseling tool. The results of the larger study have been presented elsewhere.

- In general, this is a well-written and interesting paper. The introduction is thorough and well-referenced. The introduction itself makes a great contribution to the literature, given the relatively sparse data on Niger, a country with one of the highest fertility rates in the world.
- However, the presentation of themes focuses exclusively on comparisons of several attributes across the three contraceptive methods (e.g., discretion, simplicity, familiarity etc.) The authors do not seem to consider:

1. How the context of the intervention might have influenced provider perspectives – for example, might the intervention have elevated providers’ enthusiasm for the implant relative to other methods?
2. How providers’ perspectives on the relative value of different methods might have varied by provider characteristics (e.g., age, gender or years of service).

- During analysis, did the authors explore providers’ knowledge of – and approaches to counseling about the different methods? For example, how do providers counsel about side effects, especially bleeding, as related to the three methods? How long do they counsel/expect women to use an implant before removing? What about return to fertility, side effects, etc related to oral contraceptive pills?
- In addition, given the overall lack of enthusiasm for FP in Niger, I would assume that even the providers in this study may have had some concerns about FP use among unmarried adolescents. However, the paper does not suggest any such concerns. What, if any, differences did providers make in counseling approaches for married and unmarried women?  
- Finally, the authors should provide a more nuanced understanding of how the various aspects of the three methods ranked – what themes were most and least often endorsed? For which types of users?

Specific comments:

- The methods section is well laid out and procedures for coding are best-practice. However, the authors do not describe what strategies they used beyond coding to identify their findings. For example, did the team develop any memos to get a more nuanced understanding of themes or matrices to identify any patterns of responses (perhaps any differences between perspectives of providers by age, gender, or length of service?)
- Results:
  1. Discretion: return to fertility appears to be a consideration for choosing the injectable. However, what if anything, did providers say about the impact of OCs on fertility or the potentially long period of “infertility” due to implant use? Was there any discussion about access to removal of an implant prior to its expiry if a woman wanted to get pregnant? (For a married woman who is using implants without a partner’s knowledge, does an extended period of “infertility” belie her use of a FP method?)
  2. Familiarity: some of the information provided in this section might actually fit under “simplicity” – since the insertion and removal method for implants is perceived (whether correctly or not) as more complicated and potentially painful.  Previous literature describes both the physical challenges of Norplant removal, as well as providers’ unwillingness to remove implants at the request of the user. These issues have probably been addressed over the years, but it would be useful to know whether providers in this study have confronted any challenges in removing implants.
  3. Male partner opposition to implant – in the specific example provided, what if any explanation did the provider give for the husband’s opposition to the implant?  If, for example, he also was concerned about the potential lack of discretion from implant use, it would be useful to include that perspective in the “discretion” section of the results.
- Discussion: The results section does not really provide insight into how commonly providers endorsed certain ideas. For that reason, the authors should be careful not to over interpret the qualitative findings. (For example, the inference that if women were counseled on “all methods” they would identify the implant as the best method, or the suggestion that “providers frequently discussed the misconception that women would go to hell” if they died with an implant in their arm.)

Is the work clearly and accurately presented and does it cite the current literature?

Yes

If applicable, is the statistical analysis and its interpretation appropriate?

Not applicable

Are all the source data underlying the results available to ensure full reproducibility?

Yes

Is the study design appropriate and is the work technically sound?

Partly

Are the conclusions drawn adequately supported by the results?

Partly

Are sufficient details of methods and analysis provided to allow replication by others?

Partly

Reviewer Expertise:

I am a social science researcher with expertise in qualitative and mixed method research, with a focus on acceptability, preference, adherence and use of sexual and reproductive health technologies.

I confirm that I have read this submission and believe that I have an appropriate level of expertise to confirm that it is of an acceptable scientific standard, however I have significant reservations, as outlined above.

# Response

**Published:** 2022-09-24

**Authors:** Ilene S. Speizer

**Affiliations:** University of North Carolina at Chapel Hill, USA

**Reviewer 1**

 Reviewer: In general, this is a well-written and interesting paper. The introduction is thorough and well-referenced. The introduction itself makes a great contribution to the literature, given the relatively sparse data on Niger, a country with one of the highest fertility rates in the world.

*Response: Thank you*

 Reviewer: The presentation of themes focuses exclusively on comparisons of several attributes across the three contraceptive methods (e.g., discretion, simplicity, familiarity etc.) The authors do not seem to consider:

1. How the context of the intervention might have influenced provider perspectives – for example, might the intervention have elevated providers’ enthusiasm for the implant relative to other methods?
2. How providers’ perspectives on the relative value of different methods might have varied by provider characteristics (e.g., age, gender or years of service).

*Response: The themes presented were the main themes that came out of the providers’ responses.  In the revised version of the paper, we have noted that there were no differences identified by study arm. The intervention—the segmentation tool—is meant to be a counseling tool that helps identify how to counsel clients based on various characteristics linked to an identified profile.  The segmentation strategy does not favor any method over another.*

*To address the queries by this reviewer and others, we did further analysis of the data by study arm, age, gender, years of service, and method recommendations by provider. In looking at the methods providers said they thought were most suitable for either the married or unmarried hypothetical women, there was no difference in enthusiasm or preference towards one method (e.g., implants) by study arm. Most providers mentioned the implant as the most suitable method for both women for the same reasons- length of duration, ease of use, discretion, and ability to remove at any time with a rapid return to fertility.  Overall, providers’ reasoning behind favoring implants for the unmarried woman were consistent across age, gender, and years of service.  We have included in the text a brief mention of this additional analysis. We are also limited by the number of interviews (n=24) in so far as we can ascertain demographic differences in themes that emerged by provider characteristics. This limitation has been added to the discussion.*

 Reviewer: During analysis, did the authors explore providers’ knowledge of – and approaches to counseling about the different methods? For example, how do providers counsel about side effects, especially bleeding, as related to the three methods? How long do they counsel/expect women to use an implant before removing? What about return to fertility, side effects, etc. related to oral contraceptive pills?

*Response: Information was not explored around providers’ knowledge of and approaches to counseling on the different methods and these questions were not included in our interview guide. Our study was focused on provider opinion and potential bias towards clients and method selection. That said, providers did discuss different side effects for each method. In particular, the delay in return to fertility associated with injectable use was of concern for young unmarried women who may want to quickly become pregnant if they get married. This was discussed in the paper in the section on concerns about return to fertility post method use. One of the frequently mentioned desirable attributes of the implant mentioned by providers was that it could be removed whenever needed. Return to fertility and side effects of pills were not mentioned by providers. For pills, providers spoke of the difficulty in remembering to take the pill every day, understanding how to properly take the pill (not to skip weeks of taking the pill and then restarting), and the likelihood of pills being found in a woman's belongings. In terms of side effects, pills were described as preferable for women who wanted to maintain their regular menstrual cycle. These responses all contributed to the themes that emerged in our analysis around method discretion, compliance, and return to fertility.*

 Reviewer: In addition, given the overall lack of enthusiasm for FP in Niger, I would assume that even the providers in this study may have had some concerns about FP use among unmarried adolescents. However, the paper does not suggest any such concerns. What, if any, differences did providers make in counseling approaches for married and unmarried women?

*Response: All the providers expressed clearly that their job as a provider is to present all FP methods to clients, let them make their method choice, and then administer the chosen method. Providers did not say that the unmarried adolescent should not use FP. Any concern that providers expressed around unmarried FP users was that the hypothetical unmarried adolescent should not be sexually active; however, providers said that if she was sexually active, it was better for her to use FP for pregnancy prevention as well as for STI protection than not to.  We have added this information into the text based on this and another reviewer’s inputs. The differences in counseling of married and unmarried women was one of the factors providers took into consideration when discussing which methods were best suited for each hypothetical woman.*

 Reviewer: Finally, the authors should provide a more nuanced understanding of how the various aspects of the three methods ranked – what themes were most and least often endorsed? For which types of users?

*Response: The reasons providers gave for thinking the different methods were suitable or not suitable for married and unmarried women typically fell into the theme categories and are discussed in the summary table. The rapid return of fertility associated with the implant was the only method characteristic that was noticeably mentioned more as being desirable for unmarried women. We did not feel comfortable ranking the responses by method based on the small number of interviews.*

 Reviewer: The methods section is well laid out and procedures for coding are best-practice. However, the authors do not describe what strategies they used beyond coding to identify their findings. For example, did the team develop any memos to get a more nuanced understanding of themes or matrices to identify any patterns of responses (perhaps any differences between perspectives of providers by age, gender, or length of service?).

*Response: Thank you for this comment, we have added relevant text into the methods section that describes the process in more detail.  In particular, we describe that the team developed a matrix of the emerging themes in MS Excel and discussed the themes extensively during team meetings. At times the team decided to merge themes, add new themes or take out themes based on the consensus view of the team.*

*As discussed above, no differences were observed by the characteristics of the providers.*

 Reviewer:

 Results:

- Discretion: return to fertility appears to be a consideration for choosing the injectable. However, what if anything, did providers say about the impact of OCs on fertility or the potentially long period of “infertility” due to implant use? Was there any discussion about access to removal of an implant prior to its expiry if a woman wanted to get pregnant? (For a married woman who is using implants without a partner’s knowledge, does an extended period of “infertility” belie her use of a FP method?)
- Familiarity: some of the information provided in this section might actually fit under “simplicity” – since the insertion and removal method for implants is perceived (whether correctly or not) as more complicated and potentially painful.  Previous literature describes both the physical challenges of Norplant removal, as well as providers’ unwillingness to remove implants at the request of the user. These issues have probably been addressed over the years, but it would be useful to know whether providers in this study have confronted any challenges in removing implants.
- Male partner opposition to implant – in the specific example provided, what if any explanation did the provider give for the husband’s opposition to the implant?  If, for example, he also was concerned about the potential lack of discretion from implant use, it would be useful to include that perspective in the “discretion” section of the results.

*Response: We have tried to clarify the points below in the text, when relevant and below is some explanation of what we had (or did not have) in the data.*

- *Return to fertility was discussed in the context of a long return to fertility with injectable use only. Providers did not mention return to fertility regarding OCs and implants were discussed favorably in the context of a rapid return to fertility whenever the implant was removed. There was no mention of the long period of ‘infertility’ when using an implant and neither were difficulties getting implants removed. Instead, providers spoke about how women could come get the implant removed whenever they wished to become pregnant. It is an interesting observation that women using the implant in secret may need to explain long periods of infertility; this should be researched more but did not come up in these interviews.*
- *The reason we discussed fear of implant removal in familiarity was because this fear was mentioned in terms of frightening things women had heard from others about the implant that were exacerbated by women’s overall lack of familiarity and awareness about the implant. None of the providers interviewed mentioned difficulties in removing implants or refusing to remove implants from clients requesting their removal. Implant removal was discussed in terms of clients being afraid of pain from both insertion and removal of the implant.*
- *The interviews did not include mention of the possible reasoning behind men’s opposition to the implant, only that husband opposition to implants was a reality faced by women and providers. The authors’ conjecture is that husbands also faced unfamiliarity and lack of awareness about the implant which increased their fears—this has been added to the discussion.*

Reviewer: Discussion: The results section does not really provide insight into how commonly providers endorsed certain ideas. For that reason, the authors should be careful not to over interpret the qualitative findings. (For example, the inference that if women were counseled on “all methods” they would identify the implant as the best method, or the suggestion that “providers frequently discussed the misconception that women would go to hell” if they died with an implant in their arm.)

*Response: We have revised the discussion section with this comment in mind and have tried to avoid over-interpretation of results.*
